# Automation of Controlled/Living Radical Polymerization

**DOI:** 10.1002/aisy.201900126

**Published:** 2019-12-03

**Authors:** Matthew Tamasi, Shashank Kosuri, Jason DiStefano, Robert Chapman, Adam J. Gormley

**Affiliations:** Department of Biomedical Engineering, Rutgers, The State University of New Jersey, Piscataway, NJ 08854, USA; Department of Biomedical Engineering, Rutgers, The State University of New Jersey, Piscataway, NJ 08854, USA; Department of Biomedical Engineering, Rutgers, The State University of New Jersey, Piscataway, NJ 08854, USA; Australian Centre for Nanomedicine (ACN) and the Centre for Advanced Macromolecular Design (CAMD), School of Chemistry, UNSW Sydney, Kensington, NSW 2052, Australia; Department of Biomedical Engineering, Rutgers, The State University of New Jersey, Piscataway, NJ 08854, USA

**Keywords:** automation, high throughput, oxygen tolerant, polymers, reversible addition–fragmentation chain transfer

## Abstract

Controlled/living radical polymerization (CLRP) techniques are widely utilized to synthesize advanced and controlled synthetic polymers for chemical and biological applications. While automation has long stood as a high-throughput (HTP) research tool to increase productivity as well as synthetic/analytical reliability and precision, oxygen intolerance of CLRP has limited the widespread adoption of these systems. Recently, however, oxygen-tolerant CLRP techniques, such as oxygen-tolerant photoinduced electron/energy transfer–reversible addition–fragmentation chain transfer (PET–RAFT), enzyme degassing of RAFT (Enz-RAFT), and atom-transfer radical polymerization (ATRP), have emerged. Herein, the use of a Hamilton MLSTARlet liquid handling robot for automating CLRP reactions is demonstrated. Synthesis processes are developed using Python and used to automate reagent handling, dispensing sequences, and synthesis steps required to create homopolymers, random heteropolymers, and block copolymers in 96-well plates, as well as postpolymerization modifications. Using this approach, the synergy between highly customizable liquid handling robotics and oxygen-tolerant CLRP to automate advanced polymer synthesis for HTP and combinatorial polymer research is demonstrated.

## 1. Introduction

Automated processes have been used in the laboratory for many decades to increase productivity while improving synthetic/analytical reliability and precision.^[1]^ In the pharmaceutical industry, for example, automation has significantly enabled high-throughput (HTP) tools for drug screening and the modern drug discovery process. In modern laboratories, automation has permeated almost all synthetic and analytical techniques, including autosamplers, fraction collectors, peptide synthesizers, plate readers, liquid handlers, DNA/RNA sequencing, and so on. Delegation of these highly repetitive and otherwise manual tasks significantly increases productivity and experimental throughput. This has led to HTP and combinatorial experimentation for both screening and structure–function evaluation in many fields including chemistry.

Given the wide use of polymers in advanced materials, their continued development is crucial to new technologies. In biology and medicine, polymers have found applications in drug delivery, tissue engineering, and protein stabilization. Common to all these fields is an interest in material function relationships that link the vast array of polymer compositions with specific chemical and physical properties.^[2]^ To elucidate the relationships between structure and function, HTP techniques have been widely used to explore the design of materials where theory and intuition alone have been insufficient guides to uncover mechanisms behind material performance. Such techniques use methods to screen a defined parameter space for a property of interest and utilize “hits” to drive the discovery process.

While screening enables the investigation of new materials, it also increases the demand on laboratory personnel to generate an ever-expanding library of synthetic compositions. Because of this, polymer scientists have begun to leverage automation to answer these questions. A few automated parallel synthesizers have been used to assist with automated production of polymer libraries to date,^[2b,3]^ including the Chemspeed Accelerator (SLT106, SLT II, ASW2000, SwingSLT, Autoplant A100, and SLT100),^[4]^ the Symyx system,^[5]^ and Freeslate ScPPR.^[6]^ Most of these systems allow for highly parallelized synthesis of a wide range of polymer chemistries with reasonable combinatorial throughput (up to 192 reactions for the ASW2000).

In the past, the oxygen intolerance of CLRP had limited the widespread adoption of these systems. Reaction vessels required large sample volumes (>10 mL) and inert atmosphere. In most cases, inert conditions were provided by either placing whole synthesizers in glove boxes,^[3a,b]^ sparging with inert gas,^[4e,7]^ or parallel freeze-pump-thaw cycling.^[8]^ These techniques for removing oxygen are challenging and fraught with error if not handled with extreme care. This practical limitation also requires multiple dedicated solvent dispensing lines similar to those seen in peptide synthesizers and limits the overall utility of standard liquid handlers for this type of synthesis. However, many of these issues can now be resolved by the development of oxygen-tolerant CLRP.^[9]^ Several polymerization methods have been shown to provide remarkable tolerance to oxygen in open vessels such as well plates including enzyme degassing of reversible addition–fragmentation chain transfer (Enz-RAFT),^[10]^ air-tolerant atom-transfer radical polymerization (ATRP),^[11]^ and photoinduced electron/energy transfer–RAFT (PET–RAFT) polymerization.^[12]^ By enabling CLRP in open well plates at low volume, combinatorial polymer synthesis is possible without complicated degassing and dispensing equipment.^[10b,12e,g,13,14]^

**Figure 1.**
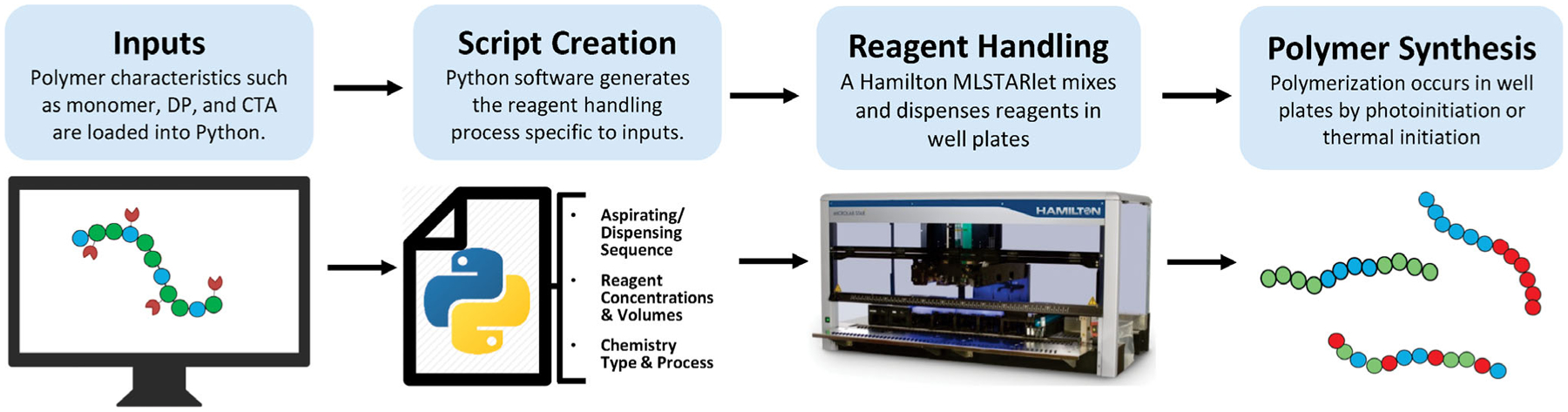
Schematic of automated process for PET–RAFT and Enz-RAFT. “Python” and the Python logos are trademarks or registered trademarks of the Python Software Foundation, used with permission from the Foundation.

The application of liquid handling robotics to prepare polymer libraries using oxygen-tolerant CLRP techniques is just beginning to emerge. Thang and co-workers recently applied Enz-RAFT to prepare polymer libraries in a Chemspeed SLT-Accelerator with 13 mL reaction volumes.^[15]^ However, moving from large reaction vessels to well plates enables a larger number of reactions to occur in parallel. We hypothesized that a highly programmable well plate-based liquid handling robot would enable greater versatility in synthetic polymerization procedures and postpolymerization handling. In this work, we present the use of a Hamilton Microlab STARlet coupled with custom Python scripts to carry out automated polymerizations using PET–RAFT and Enz-RAFT. Python was used to create custom polymer design software, which exports information about reagent species, concentrations, volumes, chemical process, and aspirating/dispensing sequences to the liquid handler. With these specifications uploaded, reagent combinations were conducted in well plates by the liquid handling robotics followed by polymerization. In addition, advanced synthetic procedures such as the synthesis of block copolymers and postpolymerization functionalization with strain-promoted azide-alkyne cycloadditions (SPAAC) click chemistry, were automated. With the transition to a robotic platform for CLRP, routine and advanced synthetic procedures may be fully automated, enabling a larger breadth of polymer material to be synthesized and explored (Figure 1).

## 2. Results and Discussion

Recent advances in liquid handling robotics have created a wide variety of instrumentation options to assist in reagent handling and aspiration/dispensing procedures. Current top-end automation equipment, such as TECAN’s Fluent and Hamilton’s Microlab, offer many advantages for HTP synthesis such as modular functionalities, automated experiment workflows, and smart reagent handling. In addition, highly customizable platforms enable the ability to interact with liquid handling robotics via programming languages such as C^++^, Python, and MATLAB. This interplay between software and hardware enables scripts to preprocess information and assist in the design of experimental workflows, which can then be conducted entirely unattended.

To explore the compatibility between automation and oxygen-tolerant CLRP reactions, Python scripts were generated to create automated systems for directing our Hamilton MLSTARlet liquid handling robot. To design these scripts, we programmed routines for PET–RAFT and Enz-RAFT chemistries based on the literature.^[10a,12c]^ These scripts took user input for desired polymer material composition, degree of polymerization (DP), and chemistry, and output for the corresponding reagent list, volumes, and dispensing sequence for the Hamilton MLSTARlet.

### 2.1. Robotic Oxygen-Tolerant PET–RAFT

**Figure 2.**
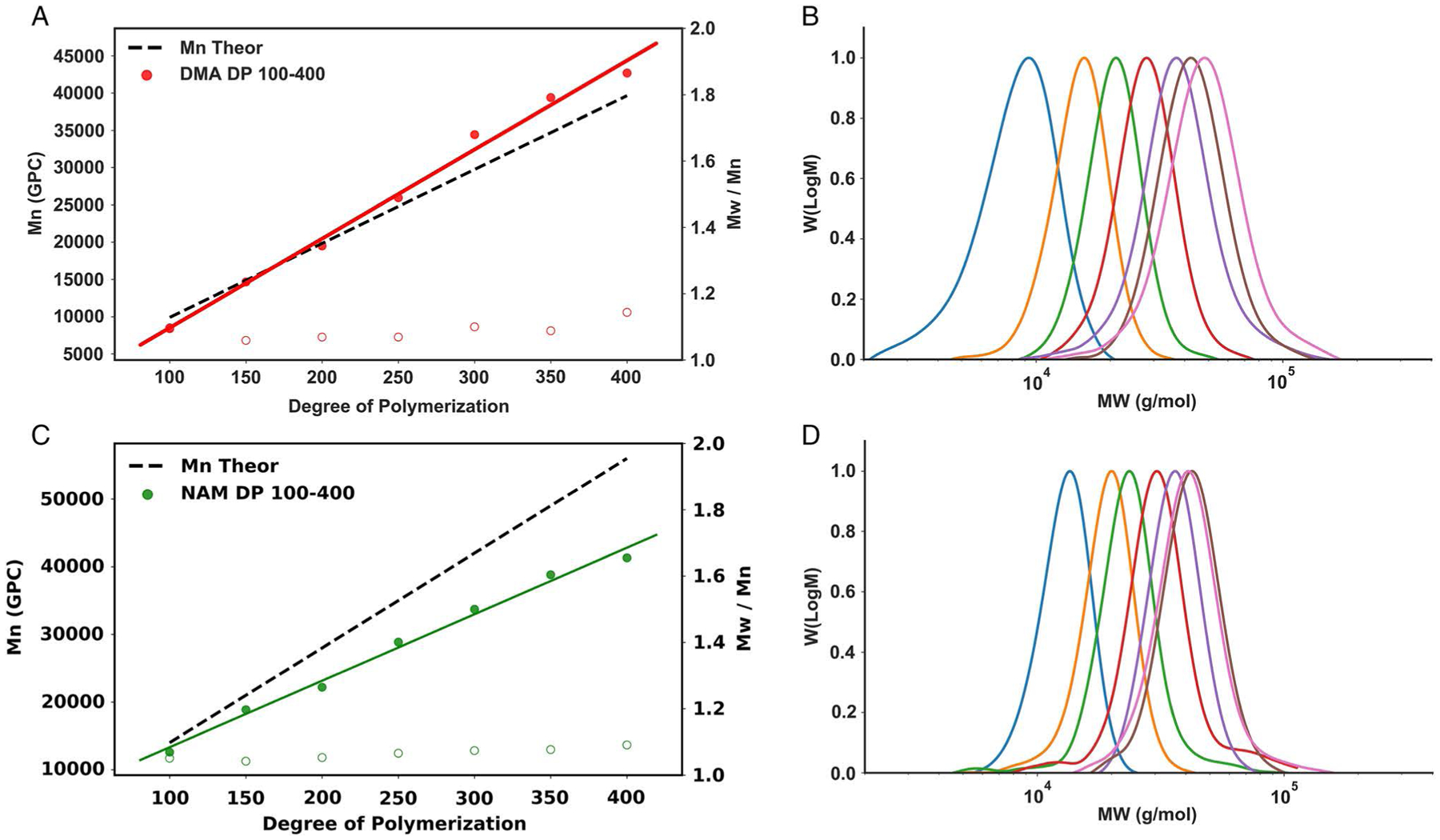
Robotic PET–RAFT. A,C) GPC-measured *M*_n_ and *Đ* of NAM and DMA polymerizations prepared by liquid handling robotics in 96-well plates. Polymerizations were irradiated under 560 nm LED light (5 mW cm^−2^) for 5 h and DP was varied from 100 to 400. B,D) Corresponding molecular weight distributions as measured by GPC.

**Figure 3.**
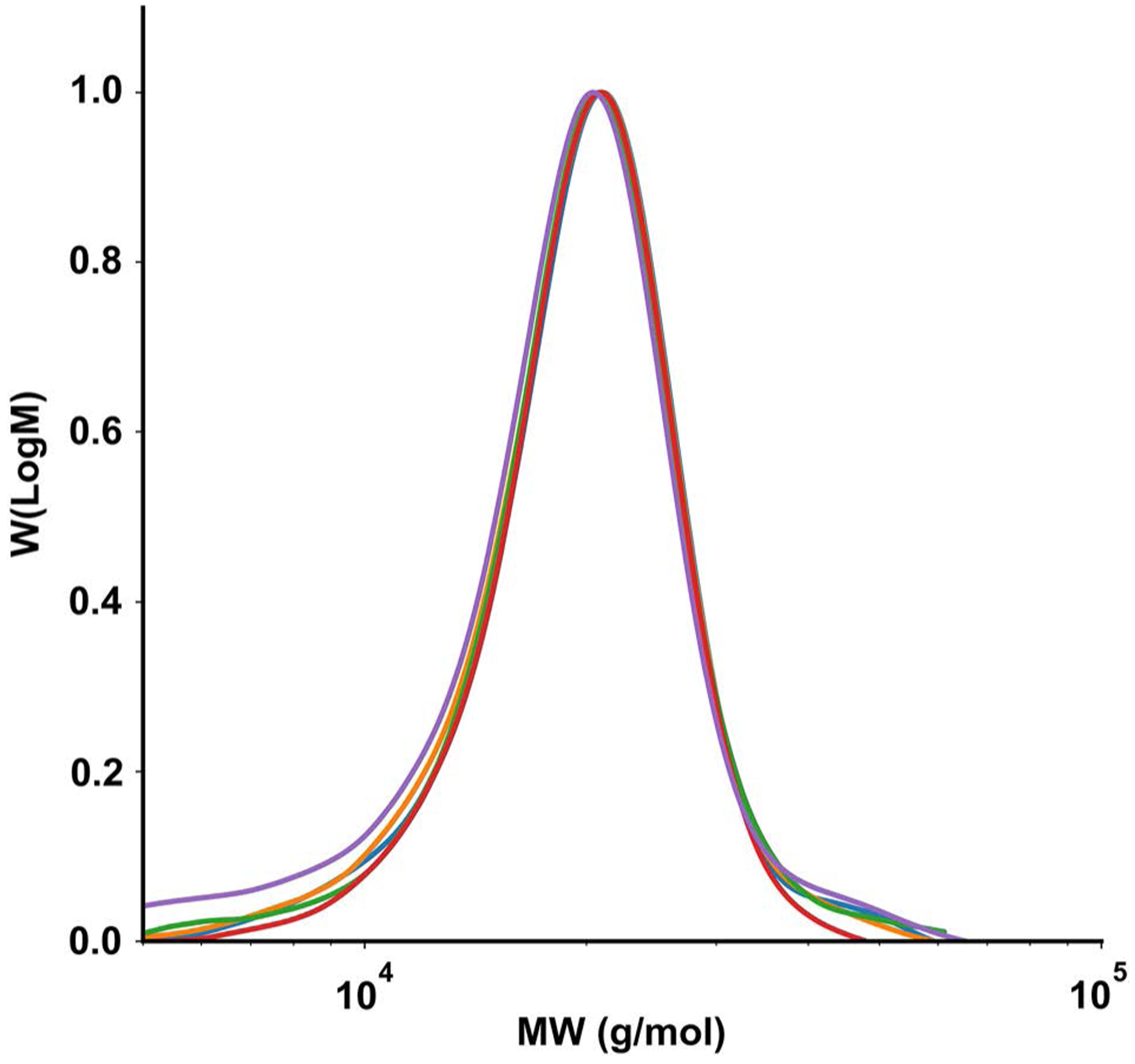
Molecular weight distributions as measured by GPC for five synthesized NAM 200 samples. Polymerizations were irradiated under 560 nm LED light (5 mW cm^−2^) for 5 h and DP was held at a constant 200/1 Mon/CTA ratio.

We began by investigating oxygen-tolerant PET–RAFT polymerizations. Linear homopolymers of *N*-acryloylmorpholine (NAM) and *N*,*N*-dimethylacrylamide (DMA) were prepared robotically in 96-well plates by PET–RAFT utilizing ZnTPP as the photoinitiator in dimethyl sulfoxide (DMSO). The monomer concentration was fixed at 1 m and the monomer/chain-transfer agent (CTA) ratio was varied from 100/1 to 400/1 to investigate the ability of the system to access a range of polymer molecular weights. Reagent aliquots were loaded into the Hamilton liquid handling robot which handled all volumetric transfers to individual wells in a 96-well plate where samples were made in parallel. After reagent transfers were completed, solutions were transferred by the robotic platform to 560 nm light-emitting diode (LED) light for 5 h to polymerize to full conversion. Excellent control over the DP and correspondingly polymer molecular weights was observed (Figure 2 and Table S1, Supporting Information). NAM and DMA polymers showed strong linear relationships between desired DP and *M*_n_, demonstrating uniform molecular weight shifts for corresponding increases in DP. While DMA demonstrated molecular weights very similar to theoretical *M*_n_, NAM demonstrated molecular weights slightly under its predicted values. As NMR shows high conversion for all NAM samples (>85%), this is likely due to inherent challenges with conventional gel permeation chromatography (GPC) calibration. In addition, both NAM and DMA polymers demonstrated low dispersity (*Đ* < 1.09 and *Đ* < 1.14, respectively), indicating excellent reaction control and amenability to an automated environment. In addition to looking at the ability of our robotic platform to synthesize and control molecular weight, we investigated the reproducibility of the system. Automated platforms are beneficial not only for their ability to scale and produce samples, but also for their ability to be consistent in the synthesis process. For this study, five independent pNAMs at DP 200 were prepared. Monomer concentration was held at 1 m and the monomer/CTA ratio was held at 200/1. All five pNAM_200_ polymers demonstrated very similar conversion, *M*_*n*_, and molecular weight distributions (Figure 3 and Table S2, Supporting Information).

### 2.2. Automated Synthesis of Block Copolymers and Postpolymerization Modification

**Figure 4.**
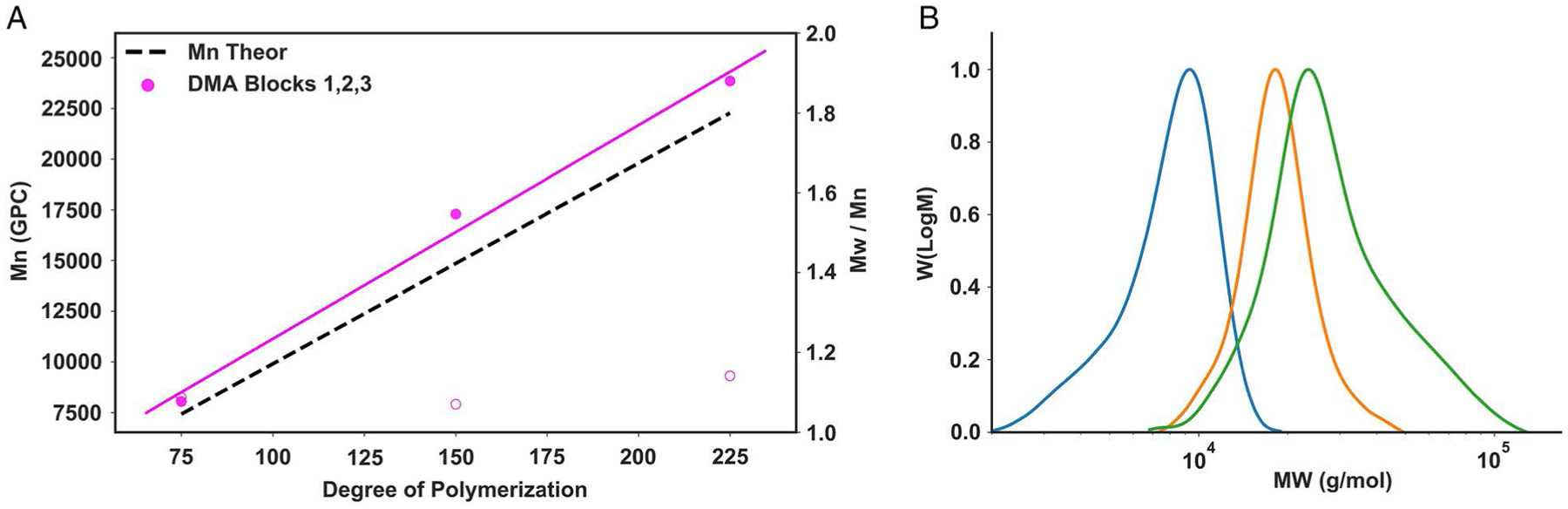
Automated synthesis of block copolymers. A) GPC-measured *M*_n_ and *Đ* of DMA tri-block copolymer prepared by liquid handling robotics in 96-well plates. DMA tri-block copolymer was irradiated under 560 nm LED light (5 mW cm^−2^) for 3 h for block 1 and 2 h for subsequent blocks. B) Corresponding molecular weight distributions as measured by GPC.

Due to the excellent chain-end fidelity of RAFT, multiple chain extensions can be performed by sequential addition of fresh monomer after near-complete conversion of the first block. Utilizing the high level of programmability in Python and the Hamilton MLSTARlet, we investigated the ability to automate the polymerization of tri-block copolymers in a 96-well plate. Tri-block *p*(DMA_75_-*b*-DMA_75_-*b*-DMA_75_) was made by repeated dispensing of monomer into the reaction well after each 3 h reaction. Clean block extensions with low dispersities (*Đ* < 1.15) were observed. Experimental molecular weights were in good agreement with the theoretical values, suggesting reaction control and successful tri-block synthesis (Figure 4 and Table S3, Supporting Information).

**Figure 5.**
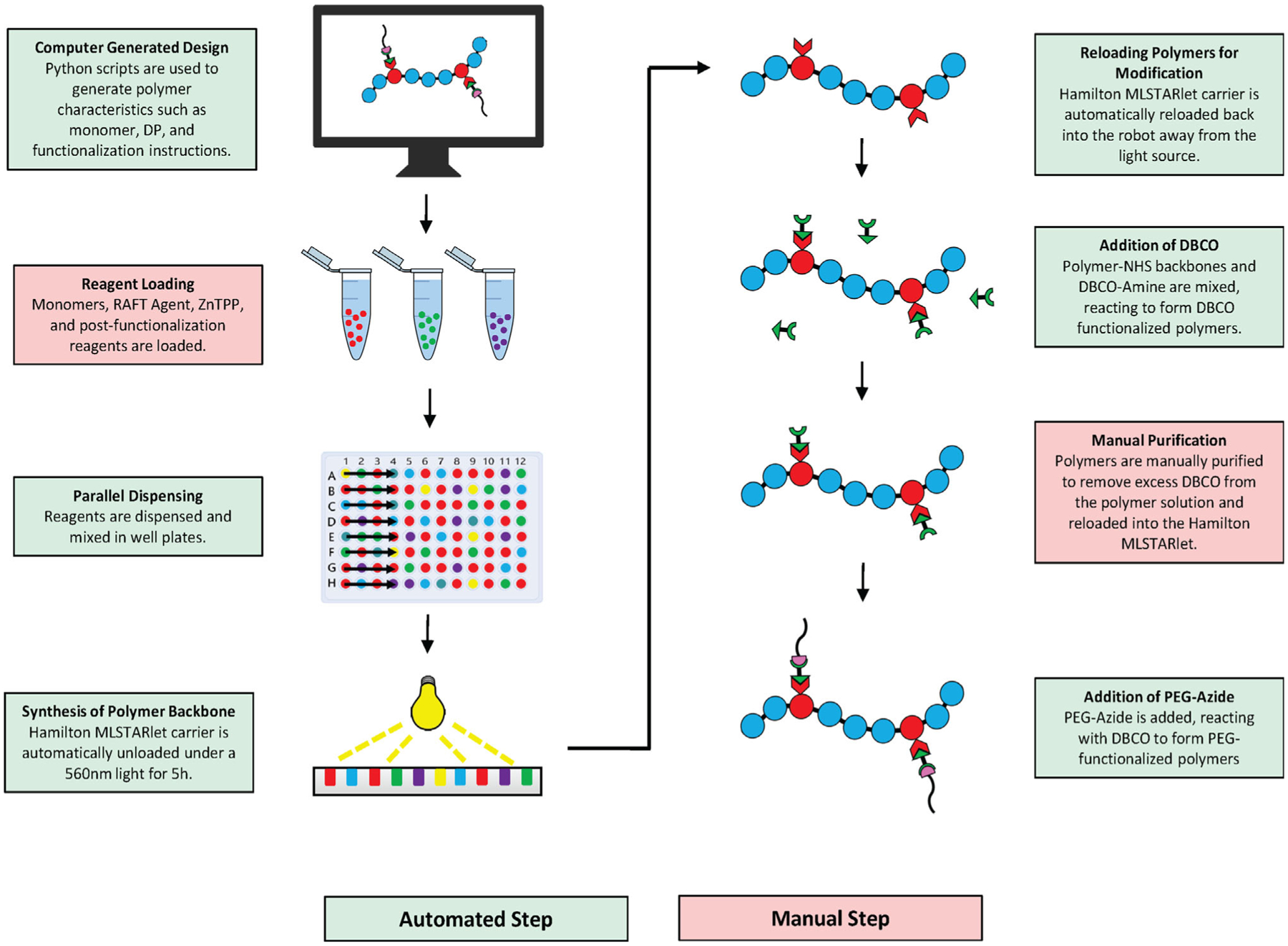
Automated synthesis and postpolymerization functionalization process.

Postpolymerization modifications are also popular tools for the generation of increasingly complex and functional materials. We hypothesized that the programmability of our robot would enable both the polymerization and functionalization steps to take place in a single automated process. To test this, we developed a reagent handling procedure for our Hamilton MLSTARlet to first synthesize polymers via PET–RAFT, and then postfunctionalize utilizing SPAAC click chemistry (Figure 5). Python scripts were used to generate aspiration/dispensing sequences for each phase of the multistep synthesis process. It also directed our Hamilton MLSTARlet to perform steps such as lid removal and replacement, carrier unloading under light for reaction initiation, and wait steps to ensure the completion of each reaction.

**Figure 6.**
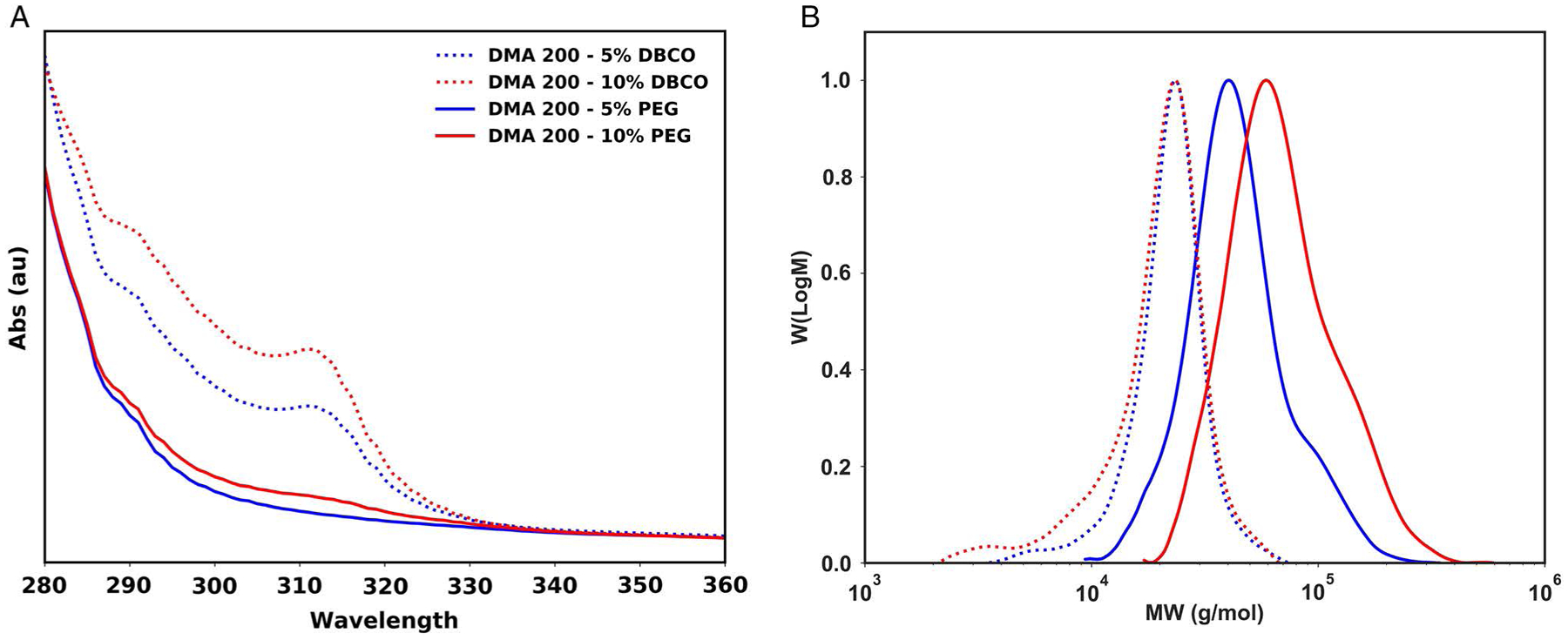
Automated synthesis and postpolymerization functionalization. A) UV–vis spectra of polymer samples showing loss of characteristic DBCO absorption peak after addition of 2 kDa PEG-N_3_. B) Molecular weight distributions for DMA/NHS polymers before and after PEG functionalization.

Linear DMA and acrylic acid *N*-hydroxysuccinimide ester (NHS-acrylate) copolymers were prepared in 96-well plates by PET–RAFT. After reagent handling by the Hamilton MLSTARlet, the 96-well plate containing the polymers was automatically unloaded by the robotic system and moved under 560 nm light to initiate polymerization. After 16 h, polymers were automatically reloaded by the system and moved back into the dispensing area. Polymers were then aspirated and dispensed to adjacent wells and mixed with dimethylaminopyridine (DMAP) and dibenzocyclooctyne-amine (DBCO-NH_2_) at a 1/1 ratio of DBCO-NH_2_/NHS followed by a 3 h wait timer to allow the reaction to complete. The Hamilton MLSTAR then prompted the user to perform a manual purification step using Sephadex-G25 spin columns to remove unreacted DBCO-NH_2_.^[16]^ UV–vis spectral absorbance (250–400 nm) of the polymer samples was collected before and after purification to determine the amount of DBCO present on the polymer. Finally, 1.0 equivalent of 2 kDa PEG-N_3_ was added to the DBCO-functionalized polymer samples and left overnight at room temperature (RT) to complete the conjugation. Automated synthesis of PEG-conjugated DMA polymers demonstrated excellent control over polymer backbone synthesis and molecular weight shifts after postpolymerization functionalization that agreed with theoretical values. At a total DP of 200, we calculated that DMA with 5% and 10% NHS-acrylate incorporation should have an average of 10 and 20 NHS-acrylate monomers, respectively, in each polymer backbone. At 100% yield, this would present expected molecular weight shifts of 20 000 and 40 000 Da with the conjugation of 2 kDa PEG-N_3_ at each site. Measured by GPC, DMA 200—5% NHS and DMA 200—10% NHS molecular weights were *M*_n_ = 21 207 (*Đ* = 1.07) and *M*_n_ = 19 194 (*Đ* = 1.13) Da, respectively (Figure 6). After functionalization with 2 kDa PEG-N_3_, these molecular weights shifted to *M*_n_ = 36 601 (*Đ* = 1.19) and *M*_n_ = 55 202 (*Đ* = 1.23) Da, respectively. These molecular weight shifts are indicative of the addition of 8 and 18 2 kDa PEG-N_3_, suggesting a 90% functionalization efficiency. These results demonstrate remarkable control of both synthesis and postpolymerization modification using an automated PET–RAFT and click chemistry process.

### 2.3. Robotic Oxygen-Tolerant Enz-RAFT

**Figure 7.**
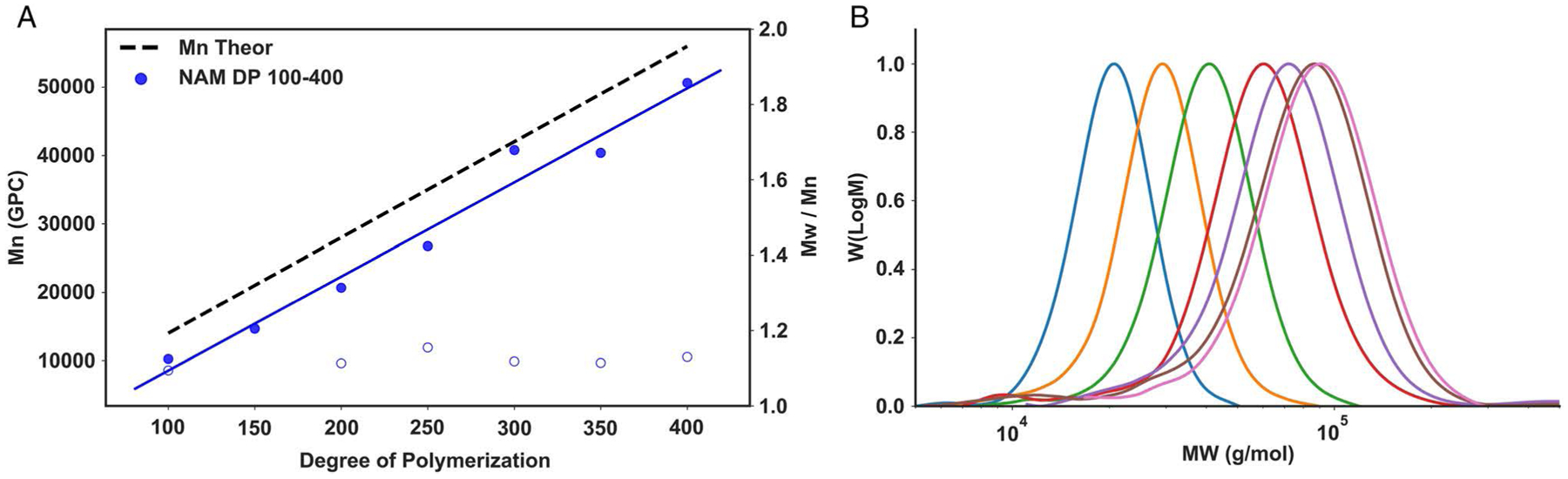
Automated synthesis by Enz-RAFT: A) GPC-measured *M*_n_ and *Đ* of Enz-RAFT prepared NAM by liquid handling robotics in 96-well plates. Polymer DP was varied from 100 to 350. B) Corresponding molecular weight distributions as measured by GPC.

We proceeded to investigate the ability of the system to also perform oxygen-tolerant Enz-RAFT polymerizations. Oxygen-tolerant Enz-RAFT was conducted by liquid handling robotics in 96-well plates to synthesize pNAM DP 100–350. Monomer concentration was fixed at 1 m, and monomer/CTA ratio was varied from 100/1 to 350/1. Reactions were conducted in 30% v/v *tert*-butanol in deionized water (DI) water saturated with glucose to allow for sufficient deoxygenation by glucose oxidase (GOx). Automated Enz-RAFT showed strong linear relationships between expected DP and theoretical molecular weights (Figure 7 and Table S4, Supporting Information). In addition, GPC-measured molecular weights were in agreement with expected *M*_*n*_ values and dispersity remained low (*Đ* < 1.2).

### 2.4. Discussion

**Figure 8.**
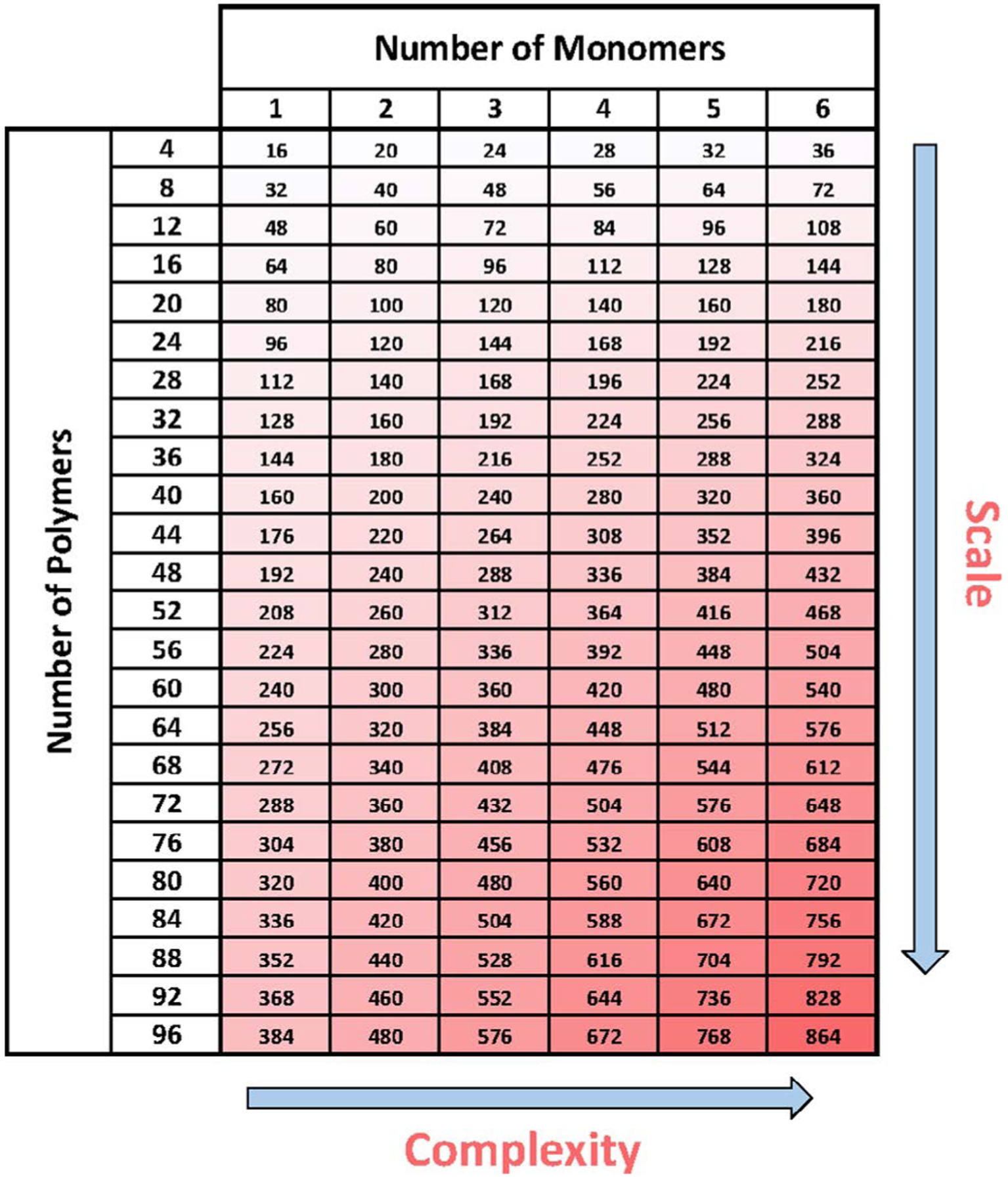
Unique transfer requirements for increased complexity and scale using PET–RAFT.

With the transition to highly versatile automation for CLRP, there exist both new challenges and new opportunities. While highly versatile liquid handling robots, such as the Hamilton MLSTARlet, provide the ability to create complex multistep synthesis procedures, they also require expertise in programming languages and hardware integration to fully automate synthetic workflows. However, once a specific chemistry has been programmed into the automation, simple graphical user interfaces (GUIs) can be developed for nonexperts to utilize the software and automation capacity without programming expertise. This can dramatically lower the barrier to entry for performing these chemistries. In addition to highly versatile liquid handlers for customized and parallel polymer synthesis, we expect the use of simpler liquid handling robotics will also prove important for HTP polymer synthesis. In one recent example by Gibson et al., a Gilson PipetMax 268 was used to prepare a library of antimicrobial polymers.^[17]^ Other systems, including Integra’s ASSIST, Hudson Robotics’ SOLO Liquid Handler, and Eppendorf’s epMotion, are examples of recent benchtop robots that can perform parallel aspiration and dispensing of multiple reagent types. While these systems do not provide the ability to fully automate synthetic workflows, they do not require extensive expertise in programming and experience in interfacing software and hardware. This allows for simpler liquid handling robots to be easily integrated into current synthesis protocols based on a laboratory’s expertise and interests. As combinatorial libraries and HTP approaches trend toward more advanced material combinations and a larger number of samples, the number of steps will rise rapidly. Highlighting this idea, PET–RAFT can be used as a model for synthesizing a complex library of random heteropolymers. Synthesizing polymers via PET–RAFT requires a minimum of four reagents: (1) monomer, (2) ZnTPP, 3) RAFT agent, and (4) DMSO. While four reagents are a minimum, synthesizing higher complexity polymers that include multiple monomers is often preferred to leverage a larger potential chemical diversity. Using this model, synthesizing 96 advanced random heteropolymers containing up to 6 different monomers requires 864 individual reagent transfers (Figure 8). While manually possible with exhaustive effort, automation enables the ability to push both the complexity and scale of combinatorial HTP approaches. To test this idea, our group developed a mock experiment to model the synthesis of 96 unique heteropolymers with 4 monomers via PET–RAFT. A member of the laboratory and the Hamilton MLSTARlet were directed to synthesize the same set of mock compounds by transferring food coloring as a marker. With this set of parameters, a total of 672 unique volume transfers were required to complete the mock library. Interestingly, while this challenged our researcher and required 3 h of intensive effort, mock synthesis by the Hamilton MLSTARlet was completed in 30 min, demonstrating >80% reduction in the time required to carry out the transfers. While our mock experiment by the laboratory personnel had very few pipetting errors over 672 transfers, it is easy to imagine mistakes are easy to occur. With liquid handling robotics, however, the probability of mistakes is kept to a minimum.

## 3. Conclusions

Coupling oxygen-tolerant CLRP with automation technologies will not only enable the field of HTP polymer synthesis to expand in both scale and complexity, but also improve chemical reproducibility. In this study, we have explored the potential for two unique oxygen-tolerant CLRP chemistries to be automated using a Hamilton MLSTARlet liquid handling robot. In particular, we show that transitioning PET–RAFT and Enz-RAFT to this platform enabled automated processes with precise control of molecular weight while maintaining low dispersity. In addition, we demonstrate the potential for a highly programmable Hamilton MLSTARlet to carry out advanced synthesis processes by the generation of a tri-block copolymer, and automated postpolymerization functionalization. With the ability to perform multiple chemistries as well as control their architecture and perform downstream modification, we believe that automated systems will play a large role in making expansive combinatorial polymer libraries and enable further HTP exploration of new materials. Going forward, we expect further automation and integration of existing and new open-air chemistries will improve access to advanced polymer materials for the nonexpert.

## 4. Experimental Section

### Materials:

Monomers *N*,*N*-dimethylacrylamide (DMA), *N*-acryloylmorpholine (NAM), and 2-hydroxyethylacrylate (HEA) were purchased from Sigma-Aldrich and deinhibited prior to use by passing them over monomerthyl ether hydroquinone (MEHQ) inhibitor removal resin. 4-cyano-4 [(dodecylsulfanylthiocarbonyl) sulfanyl]pentanoic acid, glucose oxidase (GOx) from *Aspergillus Niger*, glucose, potassium hydroxide, carbon disulfide, acetic acid, and 2-bromo-2-methylpropionic acid were purchased from Sigma, whereas zinc tetraphenylporphyrin, 1-propanethiol, and VA-044 were purchased from Fisher Scientific. Dichloromethane, ethyl acetate, and hexanes were purchased from VWR.

### Synthesis of BPTA RAFT Agent:

2-(Propyl thiocarbono thioyl thio)-2-methylpropionoic acid (BPTA) was synthesized as described in the literature.^[10a]^ Briefly, 1-propanethiol (2 g, 26.3 mmol) was added to a round bottom flask with a magnetic stir bar along with potassium hydroxide (2.94 g, 52.6 mmol) in a water/acetone mixture (2:1 v/v, 40 mL). Carbon disulfide (CS_2_, 3.32 mL, 55.3 mmol) was added dropwise and after 2 h stirring at RT, 2-bromo-2-methylpropionic acid (4.39 g, 26.2 mmol) was added and was left to stir overnight. After the removal of acetone, the aqueous solution was acidified to pH 2 with HCl and extracted with dichloromethane (2 × 50 mL). The combined organic fractions were washed with DI water (2 × 50 mL), brine (1 × 50 mL), dried over MgSO_4_, and concentrated *in vacuo*. The crude was purified using column chromatography over silica (EtOAc: hexane, 1% acetic acid), yielding the final product as a yellow-orange solid (2.5 g, 40%). ^1^H NMR (500 MHz, DMSO-*d*_6_) δ 12.69 (s, 1H), 3.27 (ddd, *J* = 8.3, 6.8, 1.0 Hz, 2H), 1.67–1.55 (m, 7H), 0.92 (tt, *J* = 7.4, 0.9 Hz, 2H).

### Polymer Characterization:

Polymer molecular weight (*M*_*w*_ and *M*_*n*_) and polydispersity (*Đ*) were measured by GPC using an Agilent 1260 Infinity II. Polymer samples were eluted through a Phenomenex 5.0 μm guard column (50 × 7.5 mm) preceded by two Phenomenex Phenogel columns (10^4^ and 10^3^ Å). GPC calibration was completed with Agilent PMMA standards. Polymers were prepared at 50:1 eluent/polymer ratio in DMF and filtered with a 0.45 μm PTFE filter. Polymer conversion was calculated by obtaining ^1^H NMR spectra using a Varian VNMRS 500 MHz spectrometer with mesitylene as an internal standard and processed using Mestrenova 11.0.4.

### Automation-Assisted PET–RAFT:

Stock solutions of monomer (2 m), 4-cyano-4-[(dodecylsulfanylthiocarbonyl) sulfanyl] pentanoic acid (RAFT agent, 50 mM) and ZnTPP (2 mM) were prepared in DMSO and pipetted into 1 mL aliquots. Aliquots were loaded into a Hamilton MLSTARlet liquid handling robot and automatically pipetted into 96-well clear flat-bottom well plates (Greiner bio-one). Monomer/CTA ratio was varied from 100 to 400, whereas ZnTPP/CTA remained at 0.01. Polymer mixtures were dispensed to a total volume of 300 μL and final monomer concentration of 1 m. They were then covered with a well-plate sealing tape and irradiated under 560 nm LED light (5 mW cm^−2^, TCP 12 Watt Yellow LED BR30 bulb) for 5 h.

### Automatic Preparation of PET–RAFT Block Copolymers:

Stock solutions of DMA (2 m), 4-cyano-4-[(dodecyl sulfanyl thiocarbonyl) sulfanyl] pentanoic acid (50 mM) and ZnTPP (2 mM) were prepared in DMSO and pipetted into 1 mL aliquots. Aliquots were loaded into a Hamilton MLSTARlet liquid handling robot and automatically pipetted into 96-well clear flat-bottom well plates (Greiner bio-one). Monomer/CTA ratio was fixed at 75 and ZnTPP/CTA at 0.01. PET–RAFT block copolymers were synthesized in a stepwise manner. Single-block DMA was dispensed at a total volume of 120 μL and final monomer concentration of 1 m. After the first dispense, the Hamilton MLSTARlet replaced the well plate lid and transferred the polymer under 560 nm LED light (5 mW cm^−2^) for 3 h. After 3 h of irradiation, the Hamilton MLSTARlet removed the plate from the light source and removed the lid to add an additional 60 μL of DMA monomer. The lid was replaced, and the sample was transferred back to the light source for another 3 h. This step was repeated again for the addition of a third block.

### Automated Postpolymerization Functionalization:

Stock solutions of monomer, RAFT agent, and ZnTPP were prepared as described earlier, whereas NHS-acrylate (2 m) stock solution was prepared in DMSO with acetic acid addition (1 equivalent/NHS) to prevent hydrolysis. All reagents were loaded into the Hamilton MLSTARlet liquid handling robot, automatically pipetted into 96-well clear plates, and polymerized under 560 nm LED light (5 mW cm^−2^) for 16 h. After polymerization, the scaffolds were functionalized with a strained alkyne (dibenzocyclooctyne–amine, DBCO-NH_2_, 1 equivalent/NHS) in the presence of DMAP (1 equivalent/NHS) and allowed to react for 3 h to incorporate DBCO onto the polymer backbone for further functionalization using SPAAC click chemistry. Unreacted DBCO was removed by spin column purification using manually packed 0.5 mL columns with Sephadex G 25 (suspended in DMSO, 37 mg mL^−1^) at 1000 rpm for 2 min.^[16]^ UV–vis absorbance scans were collected before and after DBCO purification to quantify the percentage of DBCO incorporation. After DBCO functionalization, 1 equivalent of 2 kDa PEG-N_3_/DBCO was added to the polymer solution and allowed to sit overnight for conjugation. Shifts in molecular weight before and after PEG addition were quantified by GPC.

### Automation-Assisted Enz-RAFT:

Stock solutions of monomer (2 m), BPTA (RAFT Agent, 50 mM), VA-044 (2 mM), and GOx (30 μM) were prepared in 1 mL aliquots of 30% v/v *tert-*butanol in phosphate buffered saline with 100 mM glucose. Aliquots were loaded into a Hamilton MLSTARlet liquid handling robot and automatically pipetted into 96-well clear flat-bottom well plates (Greiner bio-one). Monomer/CTA ratio was varied from 100 to 350, whereas VA-044/CTA ratio remained 0.01. Final GOx concentration in the reaction mixture was maintained at 4 μM. Enz-RAFT polymer mixtures were dispensed to a total volume of 300 μL with final monomer and GOx concentrations at 1 m and 4 μM, respectively. Polymer mixtures were covered with a well-plate sealing tape and heated at 50 °C on a heated microplate shaker for 3 h.

### Generation of Hamilton MLSTARlet Code:

Hamilton MLSTARlet sequences and processes were generated from Python with sample concentration, reagent volumes, and well position. Files containing reaction information were then transferred to the Hamilton MLSTARlet to prime the robotic transfers.

## Supplementary Material

Supporting Information
