# Supplementary material for: Automation of Controlled/Living Radical Polymerization
Source: Adv Intell Syst. Author manuscript; Available in PMC 2022 May 17. (PMC9113399; doi:10.1002/aisy.201900126)
Supplement: Supporting Information [file NIHMS1801838-supplement-Supporting_Information.docx]

**Supporting Information**

**Automation of Controlled/Living Radical Polymerization**

Matthew Tamasi,^1^ Shashank Kosuri,^1^ Jason DiStefano,^1^ Robert Chapman,^2^ Adam J. Gormley*^1^

^1^Department of Biomedical Engineering, Rutgers, The State University of New Jersey, Piscataway, NJ 08854, USA

^2^Australian Centre for NanoMedicine (ACN), School of Chemistry, University of New South Wales (UNSW), Sydney, Australia 2052

**Experimental:**

**Table S1. Automated synthesis of PET-RAFT linear homopolymers in 96 well plates at target DP 100-400.**

| # | Monomer | DP_targ_ (total) | X^a^ (%) | M_n_ theo (kDa) | M_n_ GPC (kDa) | M_w_ (kDa) | Đ |
| --- | --- | --- | --- | --- | --- | --- | --- |
| 1 | NAM | 100 | 94 | 13.2 | 12.6 | 13.3 | 1.05 |
| 2 | NAM | 150 | 92 | 19.4 | 18.9 | 19.7 | 1.04 |
| 3 | NAM | 200 | 93 | 26.1 | 22.2 | 23.4 | 1.05 |
| 4 | NAM | 250 | 95 | 33.6 | 28.9 | 30.8 | 1.07 |
| 5 | NAM | 300 | 94 | 39.6 | 33.7 | 36.2 | 1.07 |
| 6 | NAM | 350 | 94 | 46.3 | 38.8 | 41.8 | 1.08 |
| 7 | NAM | 400 | 85 | 48.0 | 41.3 | 45.1 | 1.09 |
| 8 | DMA | 100 | 97 | 9.6 | 8.4 | 9.2 | 1.10 |
| 9 | DMA | 150 | 98 | 14.6 | 14.7 | 15.5 | 1.06 |
| 10 | DMA | 200 | 99 | 19.6 | 19.5 | 20.8 | 1.07 |
| 11 | DMA | 250 | 99 | 24.5 | 26.0 | 27.8 | 1.07 |
| 12 | DMA | 300 | 97 | 28.8 | 34.4 | 37.9 | 1.10 |
| 13 | DMA | 350 | 94 | 32.4 | 39.4 | 42.9 | 1.09 |
| 14 | DMA | 400 | 97 | 38.4 | 42.7 | 48.8 | 1.14 |

**Notes:** Polymerizations were conducted under a yellow LED light (λmax = 560 nm, 6.2mW/cm^2^) varying monomer/CTA ratio between 100-400 and a CTA/ZnTPP ratio of 50/1. ^a^Monomer conversion (X) was determined by H NMR spectroscopy using mesitylene as an internal standard, an initial T0 aliquot was taken; ^b^Theoretical molecular weights (M_n_ theo) were calculated using the following equation: M_n_ theo = [Monomer]/[RAFT] * X * MW_Monomer_. Polymer molecular weights (M_n_ GPC) and polydispersity (*Đ*) were determined by GPC using PMMA standards.

**Table S2. Automated repeat synthesis of PET-RAFT pNAM DP 200**

| # | Monomer | DP_targ_ (total) | X^a^ (%) | M_n_ GPC ( kDa ) | M_w_ ( kDa ) | Đ |
| --- | --- | --- | --- | --- | --- | --- |
| 1 | NAM | 200 | 98.2 | 19.4 | 20.4 | 1.05 |
| 2 | NAM | 200 | 96.8 | 19.0 | 20.2 | 1.06 |
| 3 | NAM | 200 | 97 | 19.2 | 20.4 | 1.06 |
| 4 | NAM | 200 | 98 | 19.3 | 20.5 | 1.06 |
| 5 | NAM | 200 | 97 | 18.8 | 20.2 | 1.07 |

**Notes:** Polymerizations were conducted under a yellow LED light (λmax = 560 nm, 6.2mW/cm^2^) with a fixed monomer/CTA ratio of 75 and a CTA/ZnTPP ratio of 50/1. ^a^Monomer conversion (X) was determined by H NMR spectroscopy using mesitylene as an internal standard, an initial T0 aliquot was taken; ^b^Theoretical molecular weights (M_n_ theo) were calculated using the following equation: M_n_ theo = [Monomer]/[RAFT] * X * MW_Monomer_. Polymer molecular weights (M_n_ GPC) and polydispersity (*Đ*) were determined by GPC using PMMA standards.

**Table S3. Automated synthesis of PET-RAFT tri-block DMA (**DMA_75_-*b*-DMA_75_-*b*-DMA_75_**) in 96 well plates.**

| # | Block | DP_targ_ (total) | M_n_ theo ( kDa ) | M_n_ GPC ( kDa ) | M_w_ ( kDa ) | Đ |
| --- | --- | --- | --- | --- | --- | --- |
| 1 | DMA_75_ | 75 | 7.4 | 8.0 | 8.7 | 1.09 |
| 2 | DMA_75_-*b*-DMA_75_ | 150 | 14.9 | 17.3 | 18.5 | 1.07 |
| 3 | DMA_75_-*b*-DMA_75_-*b*-DMA_75_ | 225 | 22.3 | 23.9 | 27.2 | 1.14 |

**Notes:** Polymerizations were conducted under a yellow LED light (λmax = 560 nm, 6.2mW/cm^2^) with a fixed monomer/CTA ratio of 75 and a CTA/ZnTPP ratio of 50/1. ^a^Monomer conversion (X) was determined by H NMR spectroscopy using mesitylene as an internal standard, an initial T0 aliquot was taken; ^b^Theoretical molecular weights (M_n_ theo) were calculated using the following equation: M_n_ theo = [Monomer]/[RAFT] * X * MW_Monomer_. Polymer molecular weights (M_n_ GPC) and polydispersity (*Đ*) were determined by GPC using PMMA standards.

**Table S4. Automated synthesis of Enz-RAFT linear homopolymers in 96 well plates at target DP 100-350.**

| # | Monomer | DP_targ_ (total) | X ^a^ (%) | M_n_ theo (kDa ) | M_n_ GPC ( kDa ) | M_w_  kDa ) | Đ |
| --- | --- | --- | --- | --- | --- | --- | --- |
| 1 | NAM | 100 | 90.2 | 12.7 | 13.1 | 14.2 | 1.08 |
| 2 | NAM | 150 | 83 | 17.6 | 18.5 | 19.9 | 1.08 |
| 3 | NAM | 200 | 80 | 22.6 | 25.4 | 27.8 | 1.10 |
| 4 | NAM | 250 | 84.5 | 29.8 | 35.9 | 41.6 | 1.16 |
| 5 | NAM | 300 | 90 | 38.1 | 52.4 | 61.9 | 1.18 |
| 6 | NAM | 350 | 88 | 43.4 | 54.9 | 65.1 | 1.19 |

**Notes:** Polymerizations were conducted heated microplate shaker at 50°C varying monomer/CTA ratio between 100-400 ratio and CTA/Va-044 ratio of 20/1. ^a^Monomer conversion (X) was determined by H NMR spectroscopy using mesitylene as an internal standard, an initial T0 aliquot was taken; ^b^Theoretical molecular weights (M_n_ theo) were calculated using the following equation: M_n_ theo = [Monomer]/[RAFT] * X * MW_Monomer_. Polymer molecular weights (M_n_ GPC) and polydispersity (*Đ*) were determined by GPC using PMMA standards.
